# Cutaneous and stick rabbit illusions in individuals with autism spectrum disorder

**DOI:** 10.1038/s41598-020-58536-z

**Published:** 2020-02-04

**Authors:** Makoto Wada, Masakazu Ide, Hanako Ikeda, Misako Sano, Ari Tanaka, Mayuko Suzuki, Hiromi Agarie, Sooyung Kim, Seiki Tajima, Kengo Nishimaki, Reiko Fukatsu, Yasoichi Nakajima, Makoto Miyazaki

**Affiliations:** 1 Developmental Disorders Section, Department of Rehabilitation for Brain Functions, Research Institute of National Rehabilitation Center for Persons with Disabilities, Tokorozawa, 359-8555 Japan; 2 Faculty of Informatics, Shizuoka University, Hamamatsu, 432-8011 Japan; 3 Department of Rehabilitation for Brain Functions, Research Institute of National Rehabilitation Center for Persons with Disabilities, Tokorozawa, 359-8555 Japan; 4 Information and Support Center for Persons with Developmental Disorders, National Rehabilitation Center for Persons with Disabilities, Tokorozawa, 359-8555 Japan; 5 Hospital of National Rehabilitation Center for Persons with Disabilities, Tokorozawa, 359-8555 Japan; 6 National Rehabilitation Center for Children with Disabilities, Tokyo, 173-0037 Japan; 7 Nagano University of Health and Medicine, Nagano, 381-2227 Japan

**Keywords:** Perception, Autism spectrum disorders

## Abstract

Prediction is the process by which future events are anticipated based on past events; in contrast, postdiction is the retrospective interpretation of past events based on latter, more recent events. The prediction and postdiction are suggested to be similar based on theoretical models. Previous studies suggest that prediction is impaired in individuals with autism spectrum disorder (ASD). However, it is unclear whether postdiction is also impaired in individuals with ASD. In this study, we evaluated postdiction in individuals with ASD using the cutaneous and stick rabbit illusion paradigms in which the perceived location of a touch shifts postdictively in response to a subsequent touch stimulus. We observed significant cutaneous and stick rabbit illusion in both typically developing (TD) and ASD groups; therefore, postdiction was functional in individuals with ASD. Our present results suggest that postdiction involves a different neuronal process than prediction. We also observed that the ASD group exhibited significantly larger individual difference compared with the TD group in the stick rabbit illusion, which is considered to reflect extension of body schema to external objects. We discuss implications of the individual difference among the ASD participants in the context of sports requiring interactions between the body and external objects.

## Introduction

Individuals with autism spectrum disorder (ASD) exhibit a wide variety of motor deficits, such as impairments in imitation, motor coordination^1,2^, and tool use^1^. In such individuals, atypical motor skills are highly associated with social and communicative deficits, which represent the core symptoms of ASD^2,3^. Previous studies have also indicated that autism is a disorder of prediction^4,5^. For example, motor anticipation is reduced during postural control in children with ASD^6–8^, whereas the anticipatory behaviour in which a baby opens his or her mouth in anticipation of a spoon’s approach has been impaired in infants later diagnosed with ASD^9^. Taken together, these findings suggest that some of motor deficits in individuals with ASD were characterized by impairments in prediction (e.g., reduction of anticipatory behaviours).

Sensory perception is influenced by both prediction and postdiction, the latter of which refers to the phenomenon in which perceptions of the previous sensory signal are influenced by subsequent signals^10^. Although prediction and postdiction are hypothesised to share similar processes (e.g. a Bayesian perceptual model)^11^, it remains unclear whether postdiction is impaired in individuals with ASD. We speculated that postdiction, which shares a similar process to prediction, is also affected in individuals with ASD.

In the cutaneous rabbit illusion, successive rapid taps are delivered first at one location on the skin and then at another, causing a participant to perceive illusory taps between the two locations at which the stimuli are actually delivered, as if a small rabbit hopped along the skin^12,13^. This process by which the location of the taps is moved, following stimuli application is thought to reflect the postdictive perceptual process. If postdiction of sensory signals is impaired in individuals with ASD, the cutaneous rabbit illusion would not occur.

Previously, Miyazaki *et al*.^14^ demonstrated that the cutaneous rabbit “hops out of the body”. When participants held a stick across the tips of their index fingers of both hands and successive taps were delivered to both index fingers via the stick, they reported feeling illusory taps in the space between the actual stimulus locations (i.e. along the stick; stick rabbit illusion). This finding suggests that the cutaneous rabbit illusion involves not only an intrinsic somatotopic representation, but also a representation of the extended body schema that results from body–object interactions^15,16^.

Notably, previous studies have reported that individuals with ASD rely greatly on proprioception^3,17^. For example, in a motor learning task, such as a reaching task with disturbance by a robot arm, children with ASD rely relatively more on intrinsic coordinates (identical joint rotations as compared to the target) generated from proprioception, whereas typically developing (TD) children rely relatively more on extrinsic coordinates based on external space (identical hand motions as compared to the target)^3^. In individuals with ASD who rely on intrinsic representations, the stick rabbit illusion that uses representations of the extended body schema in external space would be weakened or would even disappear, in cases where the cutaneous rabbit illusion itself remains intact.

In this study, we assumed the possibility of impairment in the postdiction mechanism itself and in the body–object interactions in individuals with ASD. To test these hypotheses, we conducted cutaneous and stick rabbit illusion tasks in TD participants and those with ASD.

## Results

**Figure 1:**
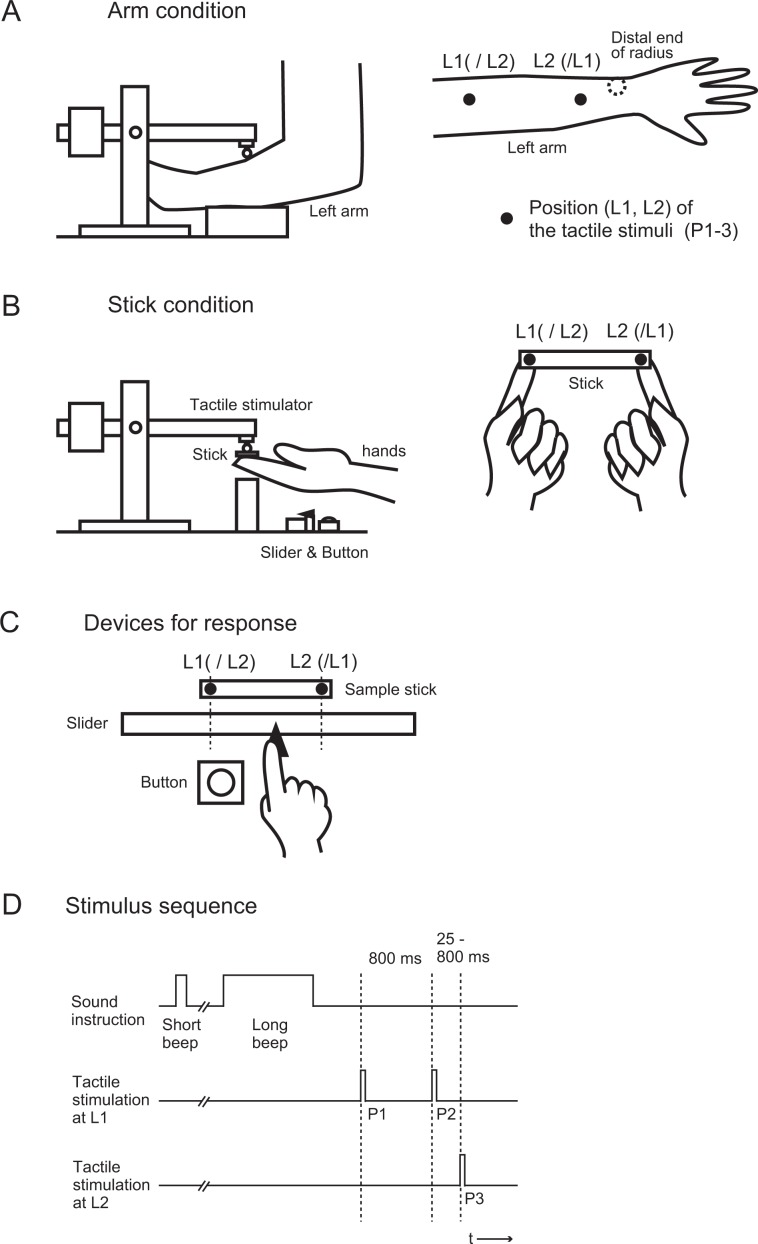
Experimental setup. (**A**) Arm condition. The stimulators were attached on the distal and proximal locations of the left arm. (**B**) Stick condition. The participant held the stick with the right and left index fingers, and the tactile stimuli were delivered to both fingers via the stick. (**C**) Devices for recording response. After the delivery of three tactile stimuli, participants opened their eyes and reproduced the locations of the three stimuli in the order in which they felt them with the reference of their left forearm (Arm condition) or using a sample stick (Stick condition), and using the slider and response button with their right hand. (**D**) Stimulus sequence. In both conditions, three tactile stimuli were delivered by a pair of stimulators. The first and second stimuli were delivered to the L1 location with a stimulus onset asynchrony of 800 ms, while the third stimulus was delivered to the L2 location with a stimulus onset asynchrony of 25–800 ms.

In each experiment, 13 participants with ASD (ASD group) and 13 TD participants (TD group) completed simple versions of the cutaneous rabbit task (Arm condition)^13,18^ and the stick rabbit task (Stick condition)^14^. In both tasks, after the delivery of the three tactile stimuli (P1, P2, and P3), the participants reproduced the locations of the three stimuli in the order in which they felt them, using the slider and response button with their right hand (Fig. 1, Supplementary Table 1). After deleting the trials in which the participants misjudged numbers or the orders of the tactile stimuli from the analysis (Supplementary Table 2), the deviations of the reported stimulus locations from the actual locations (P1, P2, and P3 deviations) were then measured.

### Cutaneous rabbit illusion in the Arm and Stick conditions in individuals with ASD

**Figure 2:**
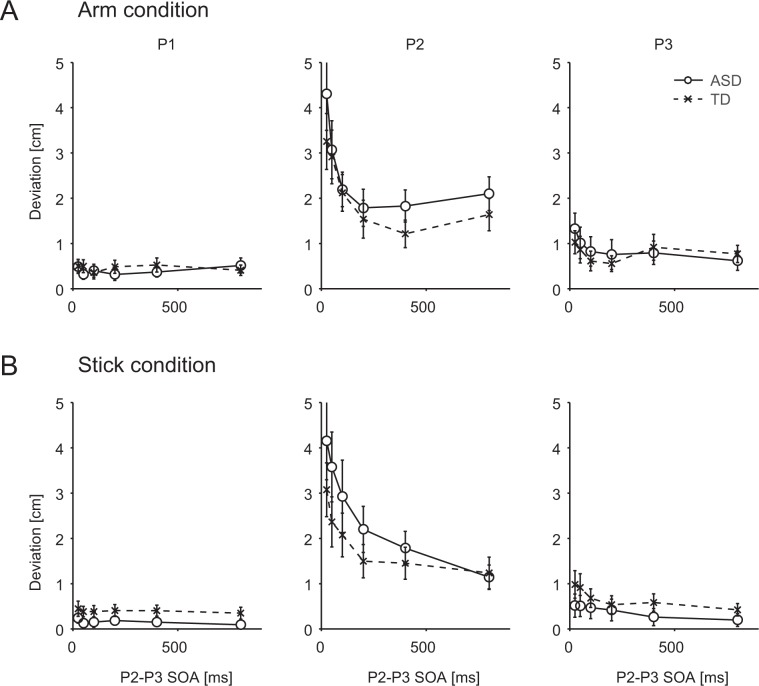
Rabbit illusions in the autism spectrum disorder (ASD) and typically developing (TD) groups. (**A**) Arm condition; (**B**) Stick condition. The abscissa indicates stimulus onset asynchronies between the second (P2) and third (P3) stimuli. The ordinate indicates the P1 (leftmost), P2 (middle), and P3 (rightmost) deviations, which reflect the degree of the rabbit illusions. Error bars indicate the standard error.

Figures 2A,B present the deviations of each stimulus (P1, P2, and P3 deviation) as functions of the P2–P3 SOA for the ASD and TD groups in the Arm and Stick conditions.

In this study, we focused on the deviation of the second stimulus (P2 deviation: perceived P2 location – actual L1 location) as a reflection of the postdictive effect^11,13,19^. In both the Arm and Stick conditions, participants in the ASD and TD groups exhibited larger P2 deviations when SOA between P2 and P3 (P2–P3 SOAs) was shorter (middle panels of Fig. 2A,B).

In the Arm condition, a two-way analysis of variance (ANOVA) with SOA (within subjects: 25, 50, 100, 200, 400, or 800 ms) and group (between subjects: ASD, TD) revealed no significant main effect of Group (*F*(1,24) = 0.59, *p* = 0.45, partial *η*^2^ = 0.024); however, we observed a significant main effect for SOA (*F*(2.71,65.14) = 15.5, *p* < 0.00001, partial *η*^2^ = 0.39). In contrast, significant interactions were not observed for Group x SOA *(F*(2.71,65.14) = 0.65, *p* = 0.57, partial *η*^2^ = 0.026). *Post hoc* analyses (Holm’s sequentially rejective Bonferroni procedure) revealed that the P2 deviation at shorter SOAs (≤100 ms) was significantly larger than that at SOAs of 400 ms (*p* < 0.05; Supplementary Table 3). In the Stick condition, a ANOVA with SOA and group revealed no significant main effect of either group (*F*(1,24) = 1.08, *p* = 0.31, partial *η*^2^ = 0.043); however, we observed a significant main effect for SOA (*F*(2.33,55.95) = 14.5, *p* < 0.00001, partial *η*^2^ = 0.38). In contrast, significant interactions were not observed for group x SOA *(F*(2.33,55.95) = 1.05, *p* = 0.37, partial *η*^2^ = 0.042). *Post hoc* analyses (Holm’s sequentially rejective Bonferroni procedure) revealed that the P2 deviation at shorter SOAs (≤100 ms) was significantly larger than that at SOAs of 800 ms (*p* < 0.05; Supplementary Table 3). These results suggest that the cutaneous and stick rabbit illusions were much more prominent at shorter P2–P3 SOAs (<100 ms), consistent with the findings of previous studies^13,19,20^. However, in the present experiments, P2 deviations (1–2 cm) were still observed even at larger SOAs (≥200 ms). We speculate that these results were due to perceptual or cognitive interference among trials, as we presented various P2–P3 SOAs within the same block.

The left panels in Fig. 2A,B present the degree of the P1 deviation (perceived P1 location – actual L1 location), which reflects errors in basic tactile localisation, free from postdiction. In each group, errors were generally small in both conditions. In the Arm condition, a two-way ANOVA with SOA and group revealed no significant main effect for P1 deviations (SOA: *F*(3.25,77.98) = 0.55, p = 0.66, partial *η*^2^ = 0.022; Group: *F*(1,24) = 0.15, *p* = 0.71, partial *η*^2^ = 0.006). There were no significant interactions between Group x SOA (*F*(3.25,77.98) = 1.08, *p* = 0.37, partial *η*^2^ = 0.043). In the Stick condition, a two-way ANOVA with SOA and group also revealed no significant main effect for P1 deviations (SOA: *F*(3.1,74.3) = 1.58, p = 0.20, partial *η*^2^ = 0.062; Group: *F*(1,24) = 2.37, *p* = 0.14, partial *η*^2^ = 0.090). There were no significant interactions between Group x SOA (*F*(3.1,74.3) = 0.10, *p* = 0.96, partial *η*^2^ = 0.042).

The right panels in Fig. 2A,B present the degree of the P3 deviation (actual L2 location – perceived P3 location). In each group, the deviations were generally small in both conditions. However, relatively larger deviations were observed at smaller SOAs in both conditions. In the Arm condition, a two-way ANOVA with SOA and group revealed no significant main effect of Group (*F*(1,24) = 0.14, *p* = 0.71, partial *η*^2^ = 0.0059). However, we observed significant main effects of SOA (*F*(3.03,72.68) = 2.82, *p* = 0.044, partial *η*^2^ = 0.11). In addition, significant interactions were not observed for Group x SOA (*F*(3.03,72.68) = 0.52, *p* = 0.67, partial *η*^2^ = 0.021). In the Stick condition, a two-way ANOVA with SOA and group revealed no significant main effect of group (*F*(1,24) = 1.03, *p* = 0.32, partial *η*^2^ = 0.041). However, we observed significant main effects of SOA (*F*(3.54,84.92) = 4.36, *p* = 0.0043, partial *η*^2^ = 0.15). In addition, significant interactions were not observed for group x SOA (*F*(3.54,84.92) = 0.64, *p* = 0.62, partial *η*^2^ = 0.026). Results of *post hoc* analyses are shown in Supplementary Table 3.

Our findings regarding the P2 and P3 deviations suggest that both the cutaneous and stick rabbit illusions were much more prominent at smaller SOAs in both the ASD and TD groups. Moreover, in the ASD group, considerable degree of the rabbit illusions were observed, compared to the trials in the larger SOA, suggesting that the process of postdiction is functional in individuals with ASD.

### Perceived P2 locations during the rabbit illusion in individuals with ASD

**Figure 3:**
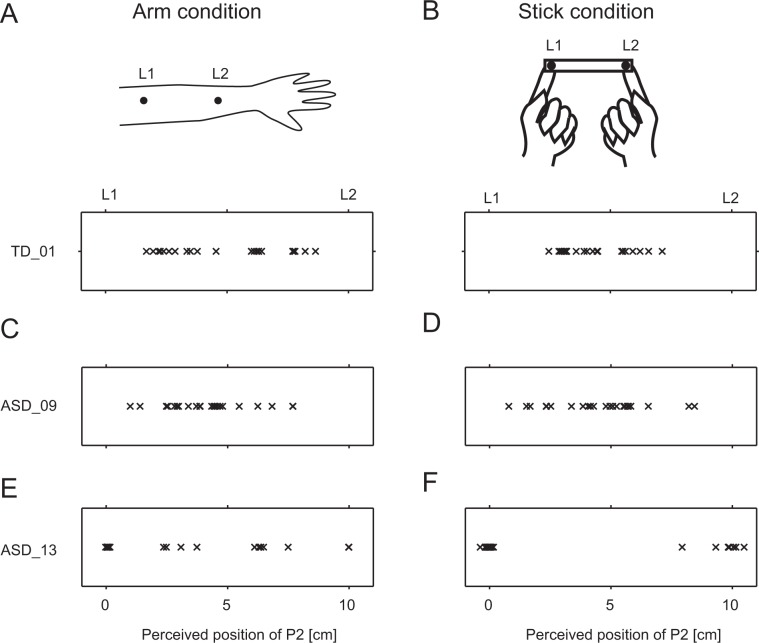
Distributions of perceived P2 locations in the Arm and Stick conditions in representative participants. (**A**) In one participant in the TD group in the Arm condition, responses suggested typical occurrence of the cutaneous rabbit illusion. (**B**) Results for the same participant in (**A**) in the Stick condition. (**C**) In one participant in the ASD group, responses were similar to those observed in the TD group for the Arm condition. (**D**) Results for the same participant in (**C**) in the Stick condition. (**E**) Another example of ASD participant’s responses in the Arm condition. (**F**) Results for the same participant in (**E**), who was biased regarding the finger locations in the Stick condition. Each mark indicates the perceived P2 location in each trial. Trials with P2–P3 stimulus onset asynchronies of 25–100 ms were used for the analysis.

In accordance with the findings of previous studies^11,13,19^, we observed that the rabbit illusions were generally more prominent for shorter P2–P3 SOAs (≤100 ms). We then examined the distributions of perceived P2 locations for trials in which P2–P3 SOAs were within 25–100 ms in each condition. This analysis revealed that there were large individual differences, especially in the Stick condition. In one participant in the TD group, the distribution of perceived P2 locations along the arm and stick suggests typical occurrence of the cutaneous or stick rabbit illusion (Fig. 3A,B). In one participant with ASD (Fig. 3C,D), the distribution of perceived P2 locations resembled that observed in TD participants. However, in the Stick condition (Fig. 3F), another participant with ASD often reported that the perceived P2 location was nearly equal to the second location (L2), where the third stimulus (P3) was actually delivered. In contrast, no such bias was observed in this participant in the Arm condition (Fig. 3E).

**Figure 4:**
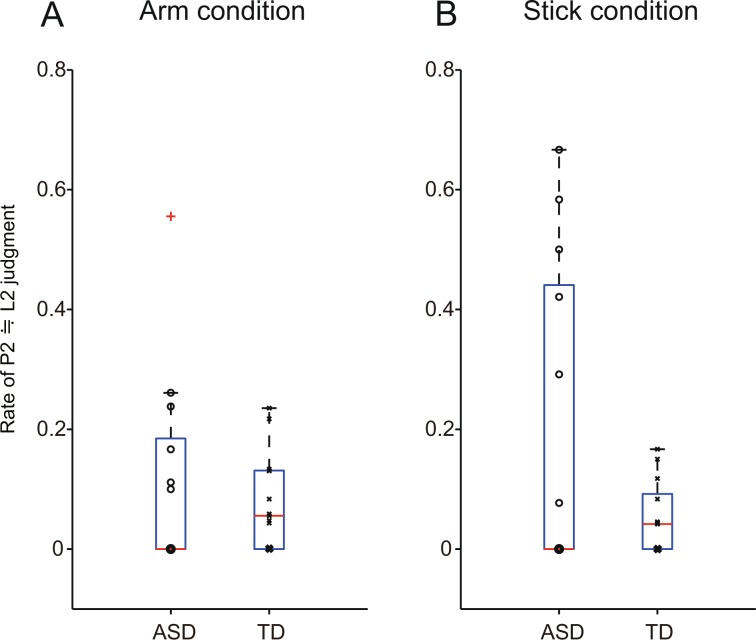
Rate of P2 ≒ L2 judgments for each participant. Box plots and individual data in (**A**) Arm condition (**B**) Stick condition. The Y-axis indicates the rate of P2 ≒ L2 judgments for each participant. Trials with P2–P3 stimulus onset asynchronies of 25–100 ms were used for the calculation. In the Stick condition, large individual differences were observed among participants with ASD. A red cross indicates an outlier. Note that multiple points overlap at each zero position in the Arm condition (ASD: 7, TD: 4) and Stick condition (ASD: 7, TD: 4).

Thus, we calculated the ratio of responses indicating that the P2 location was nearly equal to the L2 location (±10 mm) for each participant (ratio of perceived “P2 ≒ L2”) (Fig. 4A,B, and Supplementary Table 1). In the Arm condition, differences in the variations of the ratio between the ASD and TD groups did not reach significance after Bonferroni correction (*F*(11,12) = 1.56, *p* = 0.45 > 0.05/2, *Power (1-β) = *0.18, F–test), after data from one participant (indicated by a red cross in Fig. 4A) in the ASD group whose ratio was greater than +2 standard deviations (SD) away from the mean of the ASD group (0.11 ± 0.17, mean ± SD) was excluded as an outlier. In addition, compared with the distribution of the TD group, only the outlier in the ASD group showed greater ratio than +2 SD away from the mean of the TD group (0.077 ± 0.080, mean ± SD).

In the Stick condition, variations in the ratio were significantly larger in the ASD group than in the TD group (*F*(12,12) = 20.7, *p* = 0.0000073 < 0.05/2, *Power (1-β) = *0.999, F–test). Therefore, the individual differences were large in the ASD group (0.20 ± 0.26, mean ± SD). Moreover, compared with the distribution within the TD group, five of 13 ASD participants showed a ratio greater than +2 SD away from the mean of the TD group (0.056 ± 0.057, mean ± SD). That is, a subset of the ASD group often (30–70% trials in each condition) reported that the location of the second stimulus (P2) was nearly equal to the second location (L2). In contrast, such responses were not observed in the TD group. In this subset of ASD group, the cutaneous rabbit “hopped over the stick and landed on the other finger”.

We calculated the correlations between the ratio and several metrics of the participants’ properties (Supplementary Table 4). Among all participants, the age, handedness, and IQ (total IQ, verbal IQ, and non-verbal IQ) showed no significant correlation with the ratio. With regard to ADOS-2, the subscale of difficulty in imagination correlated with the ratio in both conditions.

### Relationships to daily-life difficulties

The perceived “P2 ≒ L2” ratio was not significantly correlated with Autism Diagnostic Observation Schedule-2 (ADOS-2) scores—which reflect the severity of ASD based on difficulties in social communication—in either the Arm or Stick condition (*r* = 0.32, *p* = 0.29; *r* = 0.50, *p* = 0.08, respectively). Our present results suggest a possible association between the atypical tactile perception in ASD participants and deficits in social communication; however, due to sample size and individual differences, this association was not significant.

Based on these findings, we conducted a post-survey regarding difficulties with sport-related activities in daily life. Responses were obtained from 11 of 13 participants in the ASD group. Among them, seven reported that they had difficulty with sports, including ball games. Five of these 7 participants often reported that the location of the second stimulus (P2) was nearly equal to the second location (L2) in the Stick condition. The remaining 4 participants, who did not report severe difficulty with sports, did not show such bias in the Stick condition. We speculate that the former participants represent a subset of individuals characterised by atypical tactile perception, which may explain difficulties in tool use and sports.

## Discussion

The results of the present study demonstrated that a considerable degree of the cutaneous and stick rabbit illusions was observed in the trials with shorter SOAs both in the ASD and TD groups. In contrast, to date, researchers have hypothesised that prediction and postdiction of sensory signals share similar processes (e.g. a Bayesian perceptual model)^11^. Previous studies have also suggested that ASD is associated with impairments in prediction^4^. Thus, it was plausible that postdiction is also impaired in individuals with ASD. However, our results indicated that postdiction remained functional in participants with ASD, at least in both, Arm and Stick conditions using tactile stimulation.

The differences between the prediction and postdiction might be caused by differences in regions associated with these phenomena, in the brain. Previous neuroimaging studies have reported that the premotor cortex (PMC), posterior parietal cortex (PPC), and cerebellum are involved in the prediction during the reaching movement^21^. One previous functional magnetic resonance imaging study revealed that the cutaneous rabbit illusion activates regions in the primary somatosensory cortex (S1) that correspond to the location of illusory stimulation^22^. Relevant activation was also observed in the PMC and prefrontal cortex; the authors proposed that these regions modulate S1 activity related to the cutaneous rabbit illusion^22^. Thus, the PPC and cerebellum are not activated during the cutaneous rabbit illusion. This finding suggests that postdiction is produced by a relatively simple neural network that remains intact in individuals with ASD. In accordance with this notion, prediction of low-level sensorimotor transformations also remains intact in individuals with ASD^23^.

In our study, more than one third of participants in the ASD group often reported that the location of the second stimulus (P2) was nearly equal to the second location (L2) in the Stick condition. This result indicates that while tactile postdiction functions in the ASD participants, the allocation of touch perception outside the body was difficult for these participants. The stick rabbit illusion^14^ has been interpreted as a reflection of perceptual tool–body assimilation^15,16^. Accordingly, this result may explain difficulties in tool use among individuals with ASD^24^.

Previous studies have suggested that individuals with ASD exhibit a greater reliance on proprioception than their TD counterparts. For example, in a motor learning task, such as a reaching task with disturbance by a robot arm, TD children mainly learned the reaching task using extrinsic spatial coordinates (identical hand motions as compared to the target), whereas children with ASD learned the task mainly by using intrinsic coordinates of the joints and muscles (identical joint rotations as compared to the target)^3^. In temporal order judgment (TOJ) of tactile stimuli, participants were required to answer the orders of the tactile stimuli which were successively delivered to both hands. In this task, TD participants sometimes reported reversed orders when their arms were crossed^25–27^. Such results indicate that TD participants use external coordinates rather than skin (i.e. intrinsic) coordinates during the tactile TOJ task. However, children with ASD reported fewer order reversals than TD participants^17^, suggesting that they relied on skin coordinates more than their TD peers. In the rubber hand illusion (RHI) task, synchronous brush stroking of the participant’s hand and a rubber hand demonstrated significant shifts of tactile perception to the rubber hand and produced an illusory sense of body ownership in the rubber hand^28^. The RHI is weaker or delayed in participants with ASD and in those with higher levels of autistic traits^29–31^.

Thus, earlier studies using the reaching^3^, TOJ^17^, and RHI^29,30^ tasks, revealed a preference for intrinsic coordinates in individuals with ASD. Moreover, the present study discovered difficulty with the allocation of touch perception to an external object held by the hands, in the ASD group. This result may be explained by increased reliance on proprioception in individuals with ASD. However, the large variability among participants with ASD in our study did not exist in earlier studies, suggesting that the results from the earlier studies and those from ours, do not always reflect the same phenomenon. Our results suggest that some individuals with ASD exhibit atypical characteristics of allocation of touch perception to an external object that interacts with the body. At this stage, the post-survey suggested the relationship between the atypical characteristics of allocation of touch perception and difficulties with sport-related activities in daily life. Due to the small sample size and limitation of the paradigm (i.e., the post-survey) in the present study, we could not confirm the existence of a subgroup of ASD participants that experience tactile-related difficulties with sport-related activities. Future studies with larger sample sizes and preplanned surveys are necessary to clarify the factor(s) that accounts for the individual difference in touch perception.

The PPC is thought to play an important role in the interaction between the body and external objects (i.e. in tool use and sports). For example, human neuroimaging studies have reported greater activation in the PPC during illusions related to hand–object interactions^32,33^. Moreover, one electrophysiological study involving monkeys reported that changes in the receptive fields of multisensory neurons occur in the PPC during tool use^15^. Thus, individual differences in PPC activity may be associated with the individual differences in touch perception observed in our study, which may in turn represent a biomarker for difficulties with tool use.

In addition, we found that in the Stick condition, participants with ASD occasionally misreported the orders of the first (P1) and second (P2) stimulus as compared to the participants with TD (Supplementary Table 2). In contrast to the Arm condition, bilateral fingers were used in the Stick condition, and thus, it might be explained by problems of the external spatial coordinates (i.e. left/right). Individuals with ASD might occasionally confuse spatial orientation with temporal discrepancies. We speculate that the temporal reversal of the first and second stimuli is related to the impairment in spatial working memory in individuals with ASD^34^.

In summary, this study reveals two findings. First, both in the Arm and Stick conditions, no significant difference in degree of the cutaneous rabbit illusion was observed between the ASD and TD participants. This suggests that the postdiction of touch perception was functional in individuals with ASD. Second, in the Stick condition, the cutaneous rabbit was not observed on the stick in more than one third of the ASD participants. Instead, the cutaneous rabbit “hopped over the stick and landed on the other finger” in these participants. It means that although postdiction was sufficiently functional, touch perception could not be allocated outside the body in a subset of the ASD participants. In addition, all these ASD participants reported difficulty with sports. Individual differences in touch perception of the ASD participants may explain their difficulties in sports that require sophisticated interactions between the body and external objects (i.e., equipment).

## Methods

### Participants

In this study, 13 individuals with ASD (ASD group; 1 female, 12 males; mean age of 20.4 ± 4.6 years, mean ± SD) and 13 TD individuals (TD group; 2 females, 11 males; mean age of 22.4 ± 4.4 years) participated. All participants had normal or corrected-to-normal (e.g. glasses or contacts) vision and no history of neurological diseases other than developmental disorders. The handedness of all participants was assessed using the Edinburgh Inventory (laterality quotient (LQ))^35^. All participants completed a Japanese version^36^ of the Wechsler Adult Intelligence Scale-III (WAIS-III)^37^. Since the current experiments involved minimal verbal demands, the two groups were approximately matched with respect to non-verbal IQ^34,38^ as well as age, sex, and handedness. The IQ scores of all participants were higher than 80.

Participants in the ASD group had been diagnosed with ASD, autism, or pervasive developmental disorders by medical doctors. In this study, all participants in the ASD group were administered a Japanese version of the ADOS-2^39,40^, and were classified as having autism or autism spectrum. Participant profiles are presented in Table 1 and Supplementary Table 1.

**Table 1:** Participant characteristics.

	Sex (f:m)	Age	LQ	IQ			ADOS-2 (Module 4)		
				Full	Verbal	Non-verbal	Comm.	SI	Comm. + SI
ASD	1:12	20.3 ± 1.2	72.3 ± 12	102 ± 4.3	105 ± 5.3	98 ± 3.4	3.5 ± 0.35	6.6 ± 0.62	10 ± 0.73
TD	2:11	22.4 ± 1.2	82.3 ± 6.6	115 ± 2.7	118 ± 3.8	107 ± 3.5			
	*p* = 0.56	*p* = 0.27	*p* = 0.48	*p = 0.017	*p* = 0.067	*p* = 0.069			

Each value indicates the mean and standard deviation of each metric in each group. The total IQ was slightly higher in the TD group than in the ASD group. Each value except p-values indicates mean ± standard errors. Note that the average IQ was above 100 in both groups. IQ: Intelligence Quotient, ADOS-2: Autism Diagnostic Observation Schedule Component 2, Comm.: Communication score (cut-offs: 3/2), SI: Social Interaction score (cut-offs: 6/4), Comm + SI: summed score (Communication and Social Interaction) (cut-offs: 10/7). The cut-offs mentioned above in parentheses denote the minimum scores for diagnosing ASD.

The ethics committee of the National Rehabilitation Center for Persons with Disabilities approved the study, and written informed consent was obtained from all participants prior to the experiments. All experiments were performed in accordance with relevant regulations and guidelines of Ministry of Health, Labour and Welfare of Japan.

### Apparatus

The mechanical pulses were generated by two sets of piezoelectric contactors (custom-made; Uchida Denshi, Tokyo, Japan), and each contactor was attached to the tip of a balanced pendulum (Fig. 1A,B) with the static pressure set at 2 gf. The head of the contactor was composed of acrylic material, forming a spherical body with a diameter of 6 mm. To generate each mechanical pulse, an electric pulse (50 V, 4 ms duration) was applied to the contactor, resulting in a small movement along the skin or stick surface in the transverse direction. The peak pulse amplitude was 0.035 mm in the free state. The stimulators were controlled by a programmable pulse generator (Master-8, A.M.P.I., Jerusalem, Israel) with a notebook PC that was equipped with Matlab software (Mathworks, Natick, MA, USA). The timing of the stimulation and participants’ responses were collected using a data logger (MP150, BIOPAC Systems, Inc., Goleta, CA, USA).

### Procedure for the arm condition

We conducted a simple version of the traditional cutaneous rabbit task^13,18^, which consists of three taps to the skin (Fig. 1A). The stimulators were placed on the left forearm in the horizontal direction and were located in front of the participants. The distance between the stimulators was 100 mm.

At the start of each trial, a short (200 ms) beep was presented. Then, 3,000 ms after the initial beep, a second, longer (2,000 ms) beep was presented. In response to the second beep, the participants were instructed to close their eyes and pay tactile attention to the left arm. Three tactile stimuli were then delivered to the two locations on the left arm (first location, L1; second location, L2) 500 ms after the end of the second beep (Fig. 1D).

Within the three stimuli, the first two pulses (P1 and P2) were delivered to one side (L1, proximal or distal), while the last pulse (P3) was delivered to the other side (L2, distal or proximal). In half of the trials, the order of the stimuli was “Proximal (P1)– Proximal (P2)–Distal (P3)”, while it was “Distal (P1)– Distal (P2)–Proximal (P3)” in the other half. Because the left forearm was placed in the horizontal direction, the proximal side was spatially left, whereas the distal side was spatially right. SOA between P1 and P2 was always 800 ms, while SOA between P2 and P3 varied across trials (25, 50, 100, 200, 400, or 800 ms). We repeated this block four times (2 orders × 6 SOAs × 4 times), and there were at total of 48 trials for each condition. The orders and SOAs of stimuli were randomised across trials.

After the delivery of the three tactile stimuli, participants opened their eyes and reproduced the locations of the three stimuli in the order in which they felt them with the reference of a sample stick that displayed L1 and L2, using the slider and response button with their right hand (Fig. 1C), and thereafter, the three locations were recorded in the data logger (MP150, BIOPAC Systems Inc, CA, USA). The inter-trial interval was 20 s.

### Procedure for the stick condition

In the Stick condition (Fig. 1B), participants held a wooden stick (ebony) and the stimulators with both index fingers. The distance between the stimulators was also 100 mm, and the stick was kept between the stimulators during stimulation. In this experiment, the size of the stick was 120 mm × 10 mm × 5 mm, and it weighed 3.0 g. Two holes (3 mm in diameter) were located at the end of the stick. The distance between the holes was 100 mm, corresponding to the distance between the stimulators. When participants held the stick, the centres of the finger pads were placed at the locations under the holes, and the heads of the contactors were placed over the holes. The stimulus sequence was the same as that used in the Arm condition. In response to the initial short beep, participants raised the stimulators and the stick from a pedestal. They were instructed to keep the stimulators at a height at which the plastic arms of the stimulators were approximately horizontal. In response to the second, longer beep, they were instructed to close their eyes and pay tactile attention to the entire stick. Three tactile stimuli were then delivered to the ventral pads of the right and left index fingers via the stick (first location, L1; second location, L2). In half of the trials, the order of the stimuli was “Left hand (P1)– Left hand (P2)– Right hand (P3)”, whereas it was “Right hand (P1)– Right hand (P2)– Left hand (P3)” in the other half.

After the delivery of the three tactile stimuli, participants opened their eyes and placed the stimulator and stick onto the pedestal. They then reproduced the locations of the three stimuli in the order in which they felt them with the reference of a sample stick, using the slider and response button with their right hand (Fig. 1C). The orders of the Stick and Arm conditions were counter-balanced among participants.

### Analysis

The reproduced locations of the three stimuli were stored in the data logger along with the reference positions of L1 and L2 (Fig. 1C). They were defined as the anatomical locations where the participants perceived the first, second, and third stimuli (Perceived P1, P2, and P3), and thereafter, we estimated the degree of the cutaneous rabbit effect using the reported taps (perceived P1, P2, and P3). When the participants misreported the number of stimuli (or gave no answers), the trial was excluded from further analysis (3.9 ± 1.0%, mean ± SEM). After excluding such trials, we calculated the deviations of the reported taps (Perceived P1, P2, and P3) from the actual stimulus locations (L1 and L2) to estimate the illusory effect at each SOA in both experiments (P1 deviation: perceived P1 location – actual L1 location, P2 deviation: perceived P2 location – actual L1 location, P3 deviation: actual L2 location – perceived P3 location, respectively). Among them, trials in which the P1 location was perceived to deviate from the actual location (L1) more or less than ±2 SD of the total were deemed as outliers (4.1 ± 0.81%), while the P2 and P3 deviations were evaluated as the illusory effects. In addition, the reported orders of the stimuli were occasionally reversed. We calculated the number of reversals between P1 and P2, between P2 and P3, and between P1 and P3 (Supplementary Table 2). Trials that met the criteria for outliers of the P1 deviation and for the reversed answers were excluded from further analyses.

After excluding these trials, we calculated P1, P2 and P3 deviations at each SOA of each condition in each participant. We confirmed that normality of the data was not rejected in P1, P2 and P3 deviations at each SOA using the Kolmogorov-Smirnov test (p > 0.05). We then conducted a two-way analysis of variance (ANOVA) with SOA (within subjects: 25, 50, 100, 200, 400, or 800 ms) and group (between subjects: ASD, TD) for each deviation (P1, P2, and P3 deviation) in each condition. In the test condition, according to Mendoza’s multisample sphericity test (p < 0.001), the degrees of freedom were adjusted using the Greenhouse-Geisser’s ε. After ANOVA, *post hoc* analyses were conducted using Holm’s sequentially rejective Bonferroni procedure. The data were analyzed using Matlab (MathWorks, Natick, MA, USA), R (3.3.2 GUI 1.68) with a tool to perform ANOVA (anovakun version 4.8.4) and G*Power (version 3.1.9.2).

### Follow-up survey regarding daily-life difficulties with sports

After obtaining our initial results, we conducted a follow-up survey regarding difficulties with sports during daily life, including ball games. Participants who responded that they were not good at almost all sport-related activities, including ball games, were judged to exhibit poor performance in sports. Eleven of 13 participants with ASD responded to the survey. If participants answered that they felt that they lacked proficiency in sports except for one sport, they were classified as the group who found sports difficult. Conversely, if participants answered that they thought that they were proficient in more than two fields of sports including ball games, they were not classified with those who found sports to be difficult.

## Supplementary information

Supplementary Tables 1-4.

## Data Availability

The datasets used and/or analysed in the current study are available from the corresponding author on reasonable request.
