# Supplementary material for: Cutaneous and stick rabbit illusions in individuals with autism spectrum disorder
Source: Sci Rep. 2020 Feb 4;10:1665. doi: 10.1038/s41598-020-58536-z (PMC7000771; doi:10.1038/s41598-020-58536-z)
Supplement: Supplementary file 1 — Supplementary Tables 1-4. [file 41598_2020_58536_MOESM1_ESM.docx]

**Cutaneous and stick rabbit illusions in individuals with autism spectrum disorder**

Makoto Wada^1, 2^*, Masakazu Ide^1^, Hanako Ikeda^1^, Misako Sano^3, 4, 6^, Ari Tanaka^1^, Mayuko Suzuki^5^, Hiromi Agarie^5^, Sooyung Kim^5^, Seiki Tajima^5^, Kengo Nishimaki^4, 5^, Reiko Fukatsu^3-5^, Yasoichi Nakajima^3, 7^, Makoto Miyazaki^2^

**Supplementary Table 1. Participant characteristics in detail**

Each value indicates results and metrics in each participant. Illusion [mm]: mean of P2 deviations at 25-100 ms SOAs. P2≒L2: ratio of perceived “P2 ≒ L2”. This indicates a ratio of responses that the P2 location was nearly equal to the L2 location at 25-100 ms SOAs. LQ: Laterality Quotient, IQ: Intelligence Quotient by Wechsler Adult Intelligence Scale-III (WAIS-III), AQ: Autism Spectrum Quotient, ADOS-2: Autism Diagnostic Observation Schedule Component 2, Comm.: Communication score (cut-offs: 3/2), SI: Social Interaction score (cut-offs: 6/4), Comm + SI: summed score (Communication and Social Interaction) (cut-offs: 10/7), Im: imagination score, Reps: repetitive behaviour score. The cut-offs mentioned above in parentheses denote the minimum scores for diagnosing ASD.

**Supplementary Table 2. Subjective reversals of the stimulus orders**

|  |  |  |  |  |  |  |  |  |
| --- | --- | --- | --- | --- | --- | --- | --- | --- |
|  | P1-P2 Revs [trials] | |  | P2-P3 Revs [trials] | |  | P1-P3 Revs [trials] | |
|  | Arm | Stick |  | Arm | Stick |  | Arm | Stick |
| ASD | 2.31±3.17 | 2.92±4.83 |  | 3.00±2.89 | 3.69±5.15 |  | 0.85±1.77 | 1.08±1.89 |
|  |  |  |  |  |  |  |  |  |
| TD | 2.31±2.43 | 0.15±0.38 |  | 3.38±3.84 | 1.85±2.41 |  | 0.077±0.28 | 0.92±1.26 |
|  |  |  |  |  |  |  |  |  |
|  | *p* = 0.56 | ***p* = 0.0077 |  | *p* = 0.96 | *p* = 0.37 |  | *p* = 0.55 | *p* = 0.14 |
|  |  |  |  |  |  |  |  |  |

Each value indicates the average number of reversals in each condition in each group (mean ± SD). P1-P2 Revs: number of subjective reversals between P1 and P2 stimuli; P2-P3 Revs: number of subjective reversals between P2 and P3 stimuli; and P1-P3 Revs: number of subjective reversals between P1 and P3 stimuli. Note that these trials were excluded from the analysis for evaluating the rabbit illusions.

**Supplementary Table 3. *Post hoc* test about the degree of the P2, P3 deviations**

(A) Result of *post hoc* test about the degree of the P2 deviations in the Arm condition. (B) That of the P2 deviations in the Stick condition. (C) That of the P3 deviations in the Arm condition. (D) That of the P3 deviations in the Stick condition. Each *adj. p* value indicates adjusted p-value after the correction by Holm's sequentially rejective Bonferroni procedure. Note that the P2 deviations in both Arm and Stick conditions were generally larger trials in the shorter SOAs.

**Supplementary Table 4. Correlations between the results and metrics.**

Among all participants, participant’s age, handedness and IQ (total IQ, verbal IQ, and non-verbal IQ) were neither significantly correlated with the degree of the P2 deviations nor perceived P2≓L2 ratio. As to ADOS-2 (only for the ASD participants), subscale of difficulty in imagination was correlated with the ratio in both conditions. It might be related to rigidness of thought. Conventions are same to Supplementary Table 1.
